# Telemedicine maybe an effective solution for management of chronic disease during the COVID-19 epidemic

**DOI:** 10.1017/S1463423621000517

**Published:** 2021-09-29

**Authors:** Huan Wang, Xiaojie Yuan, Jiping Wang, Chenglin Sun, Guixia Wang

**Affiliations:** 1 Department of Endocrinology and Metabolism, First Hospital of Jilin University, ChangchunJilin, China; 2 Department of Clinical Nutrition, First Hospital of Jilin University, ChangchunJilin, China

**Keywords:** chronic disease management, COVID-19 epidemic, telemedicine

## Abstract

**Aim:**

Based on the development of telemedicine and the experience of using it during the COVID-19 epidemic, we aimed to explore its convenience and shortcomings to provide a reference for the further improvement of telemedicine.

**Background:**

Traditional healthcare has been significantly affected by the outbreak of COVID-19, which has increased fear in patients with chronic diseases and increased the difficulty of obtaining hospitalized treatment.

**Methods:**

This is a conceptual article. The literature search is based on Pubmed, including articles published between January 2015 and December 2020. The purpose was to determine whether telemedicine is effective in the management of chronic diseases in the epidemic situation and to develop telemedicine and chronic disease management for long-term epidemic situations in the future.

**Findings:**

Telemedicine has demonstrated its advantages during the COVID-19 epidemic and can provide diversified clinical care services for patients with chronic diseases; these services have played a vital role in epidemic prevention and control, greatly alleviated the shortage of medical resources, increased the utilization level of medical resources, and reduced the cross-infection risk during treatment in hospitals. Furthermore, the epidemic situation presents opportunities for the development of diagnosis and treatment methods via the internet and active health management modalities.

## Introduction

Telemedicine is defined as a novel application of the internet in the healthcare industry and includes various healthcare services such as health education, medical information inquiry, electronic health records, risk evaluation of disease, online consultations, electronic prescriptions, teletherapy, and rehabilitation treatment, using the internet as both a carrier and technical means (Waller *et al.*, 2018; Weinstein *et al.*, 2018). Telemedicine, which represents a new developing direction of the healthcare industry, is conducive for solving the contradiction between the imbalance of medical resources and the increasing demand for healthcare as well as the first step of creating a smart healthcare service mode owing to the development of an information society (Dió *et al.*, 2015; Isabel de la *et al.*, 2015).

Patients with chronic diseases belong to a high-risk group during the COVID-19 epidemic because they are prone to being infected with or dying from COVID-19 (Emami *et al.*, 2020; Martini *et al.*, 2020). Because the fear of cross-infection in society has restricted their routine medical visits to hospitals, physicians should determine how to provide sufficient healthcare services for such patients (Singh *et al.*, 2020). During the COVID-19 epidemic, telemedicine is being re-examined as a medical solution to respond to the epidemic given its advantages of non-face-to-face medical treatment, no limits on time and space, and the feasibility of traceable and monitorable follow-up visits (Gadzinski *et al.*, 2020; Mahajan *et al.*, 2020). The National Health Commission of the People’s Republic of China issued *Measures for the Administration of Internet Diagnosis and Treatment (for Trial Implementation)* in 2018, according to which physicians are permitted to perform online diagnosis and treatment and issue prescriptions for subsequent online consultations for some chronic diseases. Because of the different demands and awareness of internet diagnosis and treatment in various provinces, these measures have not been implemented nationwide. In the early stage of the epidemic, medical needs and the related anxieties caused by the epidemic led to a large influx of patients into the hospital, which resulted in an insufficient supply and a weak response of offline medical resources. Additionally, subsequent measures such as the designation and transformation of fever clinics and designated hospitals as well as the suspension of clinic service have placed medical institutions in an unconventional state of operation. Because the current epidemic has made it difficult for medical services to implement their traditional hierarchy of diagnosis and treatment policies, telemedicine represents a ‘second battlefield’ for epidemic prevention and control. To alleviate the contradiction between supply and demand, as manifested by the insufficient supply of offline medical resources and the increasing number of confirmed cases, and to reduce the possibility of gathering and cross-infection, internet diagnosis and treatment services have been established in all provinces with physicians as the main workforce. Professional and technical personnel such as nurses and pharmacists are also involved (Hong *et al.*, 2020; Ren *et al.*, 2020; Ye *et al.*, 2020). According to a set of data from the Planning Division of the National Health Commission of China, during the epidemic, the number of internet diagnoses and treatments from hospitals affiliated with the National Health Commission increased by 17-fold compared with that during the same period last year. Furthermore, some third-party internet service platforms have seen an increase of >20-fold in the volume of diagnosis and treatment consultations and nearly 10-fold in the number of prescriptions compared with that during the same period last year (Feng *et al.*, 2020; Lin and Wu, 2020). Furthermore, this finding is not unique to China. In the US, where telemedicine originated in the early 21st century, the government has encouraged the replacement of offline healthcare with online healthcare since the outbreak of COVID-19 and has actively adjusted the medicare insurance policy for telemedicine accordingly; between March 2 and April 14, 2020, telemedicine consultations increased from 102.4 daily to 801.6 daily (683% increase) (Mann *et al.*, 2020). The French Ministry of Health signed a code in March 2020 that the costs of remote video consultation and remote professional service of patients with symptoms of and subsequently confirmed COVID-19 can be reimbursed by the National Health Insurance (NHI), which has helped reduce unnecessary face-to-face medical consultations, limited triage in the waiting room, increased the screening and discovery of suspected cases, etc. (Ohannessian *et al.*, 2020). Furthermore, follow-up consultations for mildly confirmed cases can be performed at the patients’ homes (JMIR Public Health Surveill., 2020). South Korea was the first country in Asia to develop telemedicine. Although this development has been affected by national healthcare policies (Lee *et al.*, 2015), the COVID-19 epidemic has prompted the country to follow the needs of the time and accept telemedicine development as a medical solution during the epidemic (Kim *et al.*, 2020). Thus, South Korea has actively adopted various policies and measures to encourage the implementation of telemedicine.

From an objective perspective, we cannot deny that telemedicine is an effective solution to the current medical dilemma and that this is an opportunity for the development of telemedicine. The epidemic has changed our lives, and more patients have begun to accept healthcare delivered through the internet. By using telemedicine to diagnose and treat patients with diabetes during the epidemic, we have discovered the feasibility of promoting this chronic disease management model in real life. In this article, we actively explore the expansion potential of telemedicine, strive to solve current and future predictable problems and address the new challenges of telemedicine.

## Advantages and opportunities of telemedicine during theCOVID-19 epidemic

### Transformation of the mode of chronic disease healthcare during the epidemic

Most patients with COVID-19 are aged between 40 and 60 years, and many of them are elderly individuals with underlying diseases or obesity (Hussain *et al.*, 2020; Wang *et al.*, 2020). Wuhan in China was the first city in which COVID-19 was discovered. Huang *et al.* (2020) published an epidemiological analysis of the first 41 confirmed cases in China in *The Lancet*, among whom 32% of the patients had chronic diseases, including diabetes, hypertension, coronary heart disease, etc. Approximately 20% of the patients had diabetes. Thus, diabetics may be a susceptible population in this epidemic because these patients have long-term hyperglycemia, which damages the blood vessels, nervous system, and other organs. In a COVID-19-related meta-analysis, Cemal Bulut evaluated eight epidemiological studies concerning COVID-19 with 46 248 confirmed cases and discovered that the most common comorbidities are hypertension, diabetes, cardiovascular diseases, and respiratory system diseases (Bulut *et al.*, 2020). Another finding of that study was that patients with these comorbidities were more likely to progress to acritical condition. According to a report from Italian Weekly, the most common comorbidities of COVID-19 are hypertension, diabetes, ischemic heart disease, and chronic renal failure, which were reported in 72%, 31.5%, 27.4%, and 23.5% of patients, respectively. This report also showed that patients without comorbidities only accounted for 2.8% of deaths (Istituto Superiore di Sanità., 2020).

Patients with chronic diseases are at high risk and are very vulnerable to this epidemic. To prevent cross-infection and infection within the hospital, patients’ visits to the hospital must be reduced to prevent exposure in this susceptible environment, and they must be provided with a home-simulated outpatient consultation environment. Three types of telemedicine platforms that provide online medical services are being promoted during this epidemic in China (Gong *et al.*, 2020; Yan *et al.*, 2020). The first type is internet hospitals operated by public hospitals. The construction of these internet hospitals is independently designed and developed by public hospitals in cooperation with other industries such as communication operators or through contracts with internet medical enterprises in various forms. From February to April of 2020, multiple internet hospitals operated by public hospitals were quickly launched in many Chinese provinces and cities. The second type of platform is internet hospitals operated by online healthcare enterprises. Online healthcare enterprises have played an indispensable role in the integration of national medical resources during the epidemic and have effectively covered areas with relatively scarce medical resources where public hospitals’ internet medical services have not yet reached. The third type is telemedicine provided on local government digital service platforms. Special services for the prevention and control of COVID-19 have been launched on most local government digital platforms, and local governments have effectively integrated the medical resources of the local online hospitals to establish a large local online hospital platform. The services provided on these online healthcare platforms include (1) fever clinic and consultation services for COVID-19; patients with confirmed COVID-19 can strengthen self-management and prevention through ‘self-check, self-inspection, and self-quarantine’ at home under professional guidance; (2) consultation services for derived health problems, particularly psychological counseling; (3) telemedicine and medical imaging teleconsultation; (4) general practice medicine (common diseases, chronic diseases): simultaneous operation of online prescription, renewal of prescriptions, and drug delivery; (5) enhancing the screening program to identify highly suspected patients to supplement the offline screening work during the epidemic; (6) special services for some projects, including fever clinics for special groups such as pediatrics/obstetrics, consulting services on nursing and nutrition, and consulting services for infection prevention and control; (7) medical assistant robot [artificial intelligence (AI)], which reduces the workload of nurses, reduces contact time with suspected and confirmed COVID-19 cases, and reduces the consumption of materials for epidemic prevention and control; and (8) detection through AI, which includes using AI thermometers to help with population screening in key places, etc. (Li *et al.*, 2020; Ye *et al.*, 2020).

The promotion of internet medical services has effectively reduced the number of outpatient visits while ensuring the demand for medical treatment. Through online contact between physicians and patients, patients have increased their knowledge and methods of prevention and self-management during the epidemic, and adverse events caused by the untimely treatment of patients with chronic diseases have been prevented (Xu *et al.*, 2020).

### Telemedicine can provide diversified clinical care services for patients with chronic diseases

Telemedicine still requires continuous improvement and expansion. Currently, telemedicine primarily aims to perfect the communication of medical information between physicians and patients and focuses on financial, goods, and other circulation issues, which is suitable for all clinical departments. However, the advantageous smart follow-up consultation functions of telemedicine for chronic diseases, such as monitoring, prevention, and clinical decision assistance, can also be extended to many other clinical departments.

The endocrinology department is the most important major chronic disease management clinical department, and diabetes is currently the most suitable disease for being treated via telemedicine (McDonnell, 2018). The self-management of diabetes includes medicines, diet, exercise, and blood glucose monitoring. The timely sharing and analysis of blood glucose data are critical for preventing hyperglycemia and hypoglycemia in diabetics (Borries *et al.*, 2019). The level of glycosylated hemoglobin and the development of diabetes complications can be predicted based on the patient’s blood glucose control level and changes in routine clinical indicators, and it is the most accurate indicator for self-management of patients (Su *et al.*, 2016). Moreover, diet and exercise can be managed by referring to the monitoring data on calorie intake and calorie expenditure. These two daily activities have a significant impact on blood glucose (Su *et al.*, 2016; Tchero *et al.*, 2019). Combining these two factors to adjust the drug can objectively reflect the actual condition of the patient. The morbidity rate of thyroid disease is increasing each year. More than 300 million individuals in the world suffer from thyroid disease, which is the second most prevalent disease in the endocrine field. Currently, approximately one in six women suffer from hypothyroidism (Dunn *et al.*, 2016). If the patient is pregnant, the probability of miscarriage and fetal mortality in the third trimester are greatly increased when the woman has thyroid disease, and impaired mental development and growth development dysfunction of the newborn baby can easily occur, causing irreparable harm to the mother and child. However, frequent offline medical visits are also unrealistic for pregnant women. Women with metabolic diseases during pregnancy require professional full-course monitoring and treatment (Akram *et al.*, 2017). Telemedicine has a unique advantage in the remote management of metabolic diseases during pregnancy (Carral *et al.*, 2015). Patients with diabetes, hypertension, and thyroid diseases are followed up through terminal monitoring equipment. In addition to interventions through the clinical nutrition department to adjust medicines for endocrine and metabolic diseases, an objective evaluation of patients’ daily metabolic balance can be achieved by combining the collection of dietary information and monitoring of calorie consumption by portable devices. This information can be used to accurately guide the nutritional follow-up of patients with chronic diseases and perform diet management in women with related metabolic diseases during pregnancy. Diabetic kidney disease develops in approximately 40% of patients who are diabetic and is the leading cause of Chronic kidney disease (CKD) worldwide (Alicic, 2017). The key to treating these diseases is to control blood glucose and blood pressure. The internet medical platform of the Endocrinology Department can be fully extended to include the management of patients in the Department of Nephrology: data on weight and serum potassium and phosphorus levels in patients could be collected to manage patients more accurately (Diamantidis *et al.*, 2018).

In addition to endocrinology-related clinical departments, coronary heart disease, hypertension, arrhythmia, and post-operative cardiovascular management in the cardiovascular medicine department are suitable for telemedicine (Omboni *et al.*, 2016; Andrès *et al.*, 2018; Song *et al.*, 2018; 2020). The technology of portable terminal monitoring equipment in the cardiovascular field—such as blood pressure meters (wireless blood pressure monitoring meter), cardiotachometers, and electrocardiogram equipment—is relatively mature and has the advantages of portability and data transmission functions (Tang *et al.*, 2018). The monitoring data, including blood pressure, heart rate, ECG, exercise volume, sleep quality, exercise heart function, etc., can be automatically uploaded to the hospital’s server through mobile phone in real time, and a real-time and follow-up monitoring service can be provided (Pineda-López *et al.*, 2018). The establishment of personalized health files based on data of patients’ cardiovascular signs allows the physicians to more objectively understand the patient’s state and progression of the illness. Respiratory diseases, including asthma, chronic obstructive pulmonary disease, and sleep apnea syndrome, are also suitable for telemedicine (Ambrosino *et al.*, 2017; Koutkias *et al.*, 2014). Portable terminal monitoring equipment such as spirometry calibration devices can be used to complement the medicines taken by patients; these devices can measure expiratory flow rate and pulmonary function index and record the frequency spectrum of breath sounds with a microphone in cell phones. These data are sent to the physician’s terminal and interpreted back to patients with a comprehensive scoring method. The physicians can use these data to analyze the patient’s condition and guide standardized treatment.

## Telemedicine experiences for diabetes during the COVID-19 epidemic

Telemedicine is not limited by time and space in real life, and it can effectively combine physicians, nurses, pharmacists, patients, patients’ families, nutritionists, and exercise therapists together into a telemedicine ecological environment to form an ecosystem of data synchronization (Hassan *et al.*, 2020). Based on the previous research experience of telemedicine (Kim *et al.*, 2016; Wang *et al.*, 2017; Sun *et al.*, 2019), we performed telemedicine for diabetes during the COVID-19 epidemic, and the research team independently developed an internet diagnosis and treatment system for diabetes on the physician’s terminal (u-diabetes.com) and the WeChatDiabetes mobile medicine system on the patient’s terminal. With the popularization of smartphones, communication between people is mostly conducted through phone applications rather than through the internet on computers (Montag *et al.*, 2015). Previous studies have shown that it is very difficult for senior citizens to learn how to use new apps. Other than surfing the internet and communicating and chatting activities, elderly patients rarely use other apps on their smartphones primarily because most apps are complicated to operate. However, if they enter the mini-program through their usual chat tool, their attitude is completely different because they have grasped the basic operation of the frequently used app. Although we have developed an independent app for patients, we still insist on collecting information from the chat tool app for the convenience of patients. Furthermore, it is much easier for patients to receive information in a timely manner without information feedback delay through this method because if you want to receive the notifications of an app, you have to first put this app on the main screen of the mobile phone. Everyone maintains a chat app on the main screen of their mobile phones. If they accidentally remove it, they will either look for it or ask others to put the chat app back on the main screen, so this ensures that the information can be fed back to the patient as soon as possible.

Considering the difficulty for elderly patients to master new things, we have created repeated online video guidance and adopted a blood glucose meter with a data transmission function (can connect directly to the smartphone through Bluetooth) to upload blood glucose data during the epidemic. Patients were asked to measure their blood glucose at least 2–3 days per week and twice a day, and they could evaluate the changes in blood glucose level, blood pressure level, and weight in the WeChatDiabetes mobile medicine system. During the epidemic, our physicians logged onto the system once every 2 weeks and adjusted the dose in a timely manner by leaving messages on the app or phone calls; these changes were made based on the data uploaded by the subjects to prevent the patients from experiencing hyperglycemia or hypoglycemia. Physicians also urged the patients to adhere to blood glucose self-monitoring as well as strengthen their diet and exercise management and register online to receive their normal treatments via the internet hospital. Nutritionists calculated the total daily calories required by referring to the original living habits of the patients, determined the composition of nutrients, and formulated recipes for three meals after converting the calories into food according to the patients’ ideal weight and nature of their work. Exercise therapists arranged regular aerobic and resistance exercises for the patients for at least 150 min a week according to different characteristics of the patients such as age, sex, physical activity, and whether they have complications.

We analyzed the patients’ control of their blood glucose without asking them to check glycosylated hemoglobin levels in the hospital because of the epidemic. From February to March, during which the epidemic was the most severe, they came to the internet hospital both to address issues with controlling their blood glucose and to consult about COVID-19 and how to prevent those with diabetes from becoming infected with COVID-19. We created a WeChat promotion video with the theme ‘Improve Immunity and Fight the Epidemic,’ which was extremely widespread with nearly 8000 clicks from the patients, likely because our internet medical platform is based on the WeChat platform. We found that the most urgent need of the patients was medical knowledge on this sudden serious emergency. Therefore, we organized human resources to provide consultations on the platform to prevent uneducated and extreme behaviors of those with diabetes and help them return to normal disease self-management. Since April, as the national epidemic situation has stabilized and normal daily life has gradually been restored in society, online medical treatment for diabetics began to increase steadily. Moreover, during the epidemic, online medical care for diabetics is not a well-planned and designed clinical research. The fasting blood glucose levels of patients with diabetes decreased by 0.37 ± 1.74 mmol/L (*P* < 0.01), and the post-prandial blood glucose levels decreased by 0.84 ± 4.37 mmol/L (*P* < 0.01) after online management for 3 months. These findings also give confidence to the therapeutic effects of online medical care on blood glucose management in diabetics in real life. Conversely, the number of patients visiting the outpatient clinic thus far has been reduced by more than 10% than in previous years. During this epidemic, the susceptibility of diabetics to infection and death has been a serious blow to patients’ desire to seek medical treatment from in-person institutions. Telemedicine is a good solution in this epidemic because it is both suitable for patients and the social environment. This epidemic is also a turning point in the transformation of the medical service mode.

## Current barriers, solutions, and future development of telemedicine

### Barriers to telemedicine popularization in clinic

Telemedicine is also a type of medical behavior; it is an online extension of the hospital’s work scenario with the main body of physicians and nurses. However, the current form of telemedicine is extremely dependent on internet technology. Although physical hospitals use technology, the knowledge system and emotional communication among people in hospitals are shaped by the experiences of medical workers who learn and work in actual hospital scenarios. Therefore, given their experience, senior medical staff should be consulted when designing the online working scenario. Without sufficient medical information collection, accurate diagnosis and treatment logic, and a rigorous error correction and prevention system, it is impossible for physicians to successfully provide medical services online.

Telemedicine has promoted the transition of chronic disease treatment from hospitalized treatment to interactive communicative remote/mobile treatment. Big data technology has been adopted to dynamically collect and compare health risk factors to identify health-related risk factors and propose targeted intervention plans through an intelligent medical decision-making system (Amato *et al.*, 2019). This system, which can be used to improve the comprehensive analysis capabilities of medical data and help physicians improve clinical decision-making accuracy, has made the integrated medical service mode of remote monitoring and intelligent diagnosis for chronic diseases an inevitable trend of social development. There is an urgent need for medical institutions to share medical diagnosis and treatment technologies and patients’ management information on scientific and technological service platforms to rapidly make innovations in the medical service mode for chronic diseases.

The collection of sufficient medical information benefits from the data transmission capabilities of various portable devices and the development of AI recognition technology; similarly, the technology of recognizing and transforming unstructured data in medical information into structured data and the technology of recognition and digital conversion of monitoring image information in life improve the collection of medical information (Ranjan *et al.*, 2019). Integration, screening, and analysis of information and other technologies for portable and home monitoring equipment must be further improved to take full advantage of telemedicine to provide a more personalized and precise diagnosis and treatment than traditional on-site medical services. Accurate diagnosis and treatment logic come from the experience of senior medical staff and cannot be replaced by clinical diagnosis and treatment manuals or diagnosis and treatment guidelines. The data and analysis process of IBM Watson come from guidelines, high-level literature, and high-level hospital experience. It is the most representative intelligent clinical decision-making assistance system. However, making clinical decisions is not as simple as playing GO/chess. Several factors affect the physical condition of the human body. In addition to disease, these factors include complications, mental conditions, etc., and determining the proper treatment cannot solely depend on written guidelines. The intelligent clinical decision-making assistance system for chronic diseases must be supported by sufficient personal medical information with the integration of dynamic data. In this process, the physician’s thinking mode must be strictly followed. A rigorous error correction and prevention system is an unsurpassable advantage of telemedicine. The entire diagnosis and treatment process should be rigorously designed because it is full of complicated situations such as differential diagnoses, the judgment of indications and contraindications, etc. Including functions of error prevention and microcomputer corrections can help guarantee telemedicine safety.

### Future development of telemedicine, an active healthcare model

Humans are facing a long-term fight against the COVID-19 epidemic, and we might encounter similar challenges again in the future. This epidemic reminds us of the necessity of establishing a healthy work and living style. With the development of big data and AI technology, passive health management is gradually becoming active health management; the trend of static retrospective analysis transitioning to dynamic, forward-looking analysis is also a characteristic of the big data era. Big data technology provides people with new methods and approaches to understand and transform the world, which is an innovation in the medical industry.

The current telemedicine system can greatly facilitate the diagnosis and treatment activities of doctors and patients, effectively assist clinical work, and realize personalized diagnosis and treatment. Telemedicine is not limited by time and space, and it can process large amounts of medical information simultaneously, which can expand the target users from patients requiring medical treatment to sub-healthy people and healthy people who only require health management (Ren *et al.*, 2019). Our current system of health management is passive, and patients accept medical intervention only when there is something wrong with their bodies. Medical interventions also target lesions to eliminate these phenomena to relieve pain or cure sickness. However, active health management is a medical mode that actively applies controllable stimulation to the human body to enhance the adaptability of the human body and thus strengthen its function or reverse chronic diseases. The human body is a complex system with strong self-healing and self-organization abilities. The task of medicine is to fully exploit the controllable methods and means for completely using the human body’s abilities to eliminate human diseases and improve the human body. This requires long-term continuous dynamic tracking to identify and evaluate the personal health state and its evolution and to perform active interventions via the comprehensive use of various medical methods to maintain the human body in a healthy state. Telemedicine meets the needs of active health scenarios because it can realize the cyclical closed-loop management among hospitals, work units, and families. Because the main scenarios of telemedicine are applicable to work units and families, the purpose of telemedicine is not only to treat diseases but also to perform prevention and treatment activities with physicians being considered the health ‘coaches’ and the patients’ friends and living styles, such as exercise and nutrition, the tools for treatment.

Currently, telemedicine focuses on establishing scenarios for serving medical institutions and medical workers and addressing key issues such as controlling health risk factors and health services for the aging population. In the future, new-generation information technologies such as mobile internet, big data, wearables, and cloud computing must be integrated to focus on the dynamic identification of status, risk assessment, and self-management of health. These technologies will be used to address the difficulties and bottleneck issues associated with the continuous and dynamic collection of health information and fusion analysis of big data on health and to build an integrated health service system led by active health technology. This will be another opportunity and challenge for telemedicine.

## Implications and conclusion

We all understand that the current COVID-19 epidemic is a global struggle between mankind and the environment, and we can receive limited help from others in the face of this major catastrophe. When people are quarantined and spaces are divided, we must take “my health, my decision” into consideration. As the most effective tool to help everyone maintain physical and mental health, telemedicine should expand its advantages from disease treatment to prevention and then even further to help maintain public health. Although the medical service mode is experiencing major changes as a result of the transition to telemedicine, a great leap in telemedicine is worth looking forward to.
